# Human Cystic Echinococcosis in Different Geographical Zones of Iran: An Observational Study during 1995–2014

**Published:** 2017-12

**Authors:** Mohammad ZEINALI, Mehdi MOHEBALI, Mohammad Reza SHIRZADI, Bahman RAHIMI ESBOEI, Hossain ERFANI, Jamshid POURMOZAFARI, Mahboube GHANBARI

**Affiliations:** 1. Dept. of Medical Parasitology and Mycology, School of Public Health, Tehran University of Medical Sciences, Tehran, Iran; 2. Center of Communicable Diseases, Ministry of Health and Medical Education, Tehran, Iran; 3. Center for Research of Endemic Parasites of Iran (CREPI), Tehran University of Medical Sciences, Tehran, Iran

**Keywords:** Cystic echinococcosis, Human, Iran

## Abstract

**Background:**

Cystic Echinococcosis (CE) is a serious zoonotic parasitic disease in Iran. This study aimed to show the trend of the confirmed disease from 1995 to 2014 and to describe some of epidemiological aspects of the disease in Iran.

**Methods:**

This retrospective study has been designed based on data collected from 8518 cases of CE among various geographical locations of Iran.

**Results:**

The average annual number of human cases of CE was 274.8. Among 31 provinces of Iran, Razavi Khorasan from northeast part of Iran was the highest human CE infected province with the 1801 cases and Hormozgan Province in south part of the country showed the lowest the disease with the only one case of CE in 2009. Liver and lungs with the infection rate of 61% and 20%, respectively are the most infected organs, 53% of patients had one cyst in the bodies and the number of cysts in 8% of cases was more than 3 cysts. Altogether, 41% of CE cases were treated by surgery, 11% with chemotherapy and 48 % with mixed surgery and chemotherapy.

**Conclusion:**

Human CE is a major health problem in Iran and it is necessary to establish basic control programs. It is crucial to setting up standard diagnostic methods for early diagnosis, effective treatment, plan educational schedule for different social levels and control the disease in both definitive and intermediate hosts.

## Introduction

Human cystic echinococcosis or human hydatidosis is a zoonotic infection caused by larval forms (metacestodes) of tapeworms of the genus *Echinococcus* spp. live in the small intestine of domestic and wild canines. Although there are different species of *Echinococcus* described, only two of them including *E. granulosus* and *E. multilocularis* are pathogenic for humans. To differentiation the diseases caused by these two different species, WHO proposed the designation Cystic Echinococcosis (CE) for the disease caused by *E. granulosus* and Alveolar Echinococcosis (AE) for the disease caused by *E. multilocularis* (1).

Human echinococcosis is a zoonotic parasitic infection caused by the larval stage of *E. granulosus* recommended to be appointed in neglected parasitic disease by WHO reports (1). Echinococcosis is a main public health concern, especially in developing countries with inadequate economic resources (1–4). Human hydatidosis is commonly asymptomatic and endurable for many times until they cause a force on neighboring tissues. They have usually diagnosed accidentally during exploration for other pathology(5). The clinical manifestations are similar to a slowly growing tumor and depend on the size, number and the site of the cyst(6). About 60%–70% of CE takes place in the liver and 20%–25% in the lungs. Bones, kidneys, spleen, muscles, CNS and behind the eye are the other organs infected by cysts(7). Dogs and other canines are the definitive hosts for *E. granulosus* that sub-clinically carry the adult form of the worm and because of the close relationships to humans are particularly important in zoonotic transmission(8). Humans, sheep, cattle, buffalo, camels, and pigs are the commonest intermediate hosts that infected by the ingest tapeworm eggs from foods and contaminated water (9). Hydatid disease is particularly common in rural regions, chiefly sheep-raising areas. The annual incidence of human CE can range from less than 1 to 200 per 100000 inhabitants in various endemic areas (10). CE is endemic in the entire Mediterranean regions including all countries from the Middle East. Hydatidosis is endemic in Iran so it must be considered as a problem. Five to 49% of stray dogs are infected with *E. granulosus* in different areas of Iran. Sheep, camels, and cattle with the prevalence rate of 88%, 70% and 10%, respectively are the main intermediate host in Iran (11). In the present study, we estimated the pattern of human CE in the Iran from 1995 to 2014.

## Methods

This retrospective study was designed using data from the national hydatidosis surveillance system. The communicable diseases control organization of the Iranian Ministry of Health is responsible for surveillance and passive collection of data of communicable diseases from various provinces of Iran. Computerized Tomography (CT) scans serology; echography and sonographic methods are mostly using in diagnosis of human CE in this study (12). Hydatidosis surveillance data from different governmental and private hospitals in all parts of country, were should record in Health Information Management. These data monthly submitted to the communicable diseases control organization of the Iranian Ministry of Health. In current work, data from 1995 to 2014 monthly gathered online from 355 districts. The data collected for this survey included individual and geographical information, laboratory data and treatment methods for all cases of human CE. The zoonosis department is a subset of the communicable diseases control organization and similar information for human CE was assessed and analyzed by this department.

Ethical approval for the study was obtained from the national hydatidosis expert committee.

The data were analyzed using SPSS software (Chicago, IL, USA, version 19.0), and Microsoft Excel 2010 at the communicable diseases control organization.

## Results

### Trends of incidence rate of human CE during 1995–2014:

**Fig. 1:**
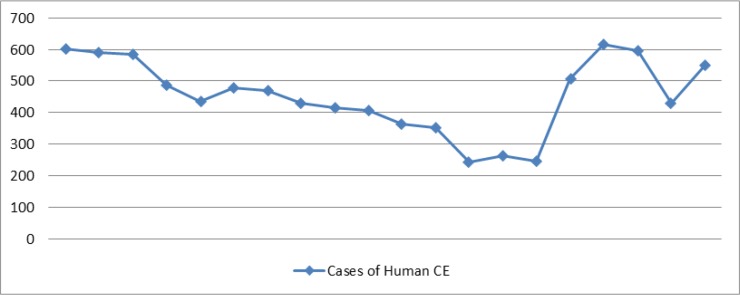
Trend of human CE in Iran, showing number of cases and incidence rate per 100 000 inhabitants, 1995–2014

The data on all types of echinococcosis in Iran were collected from 1995 to 2014. Over this period, the total number of cases reached 8518. The trend in cases of human CE from 1995 to 2014 is illustrated in Fig. 1, showing the number of the cases in each year. The highest incidence rate of echinococcosis was reported in 2010 with 615 cases and the lowest in 2007 with 246 cases.

**Fig. 2:**
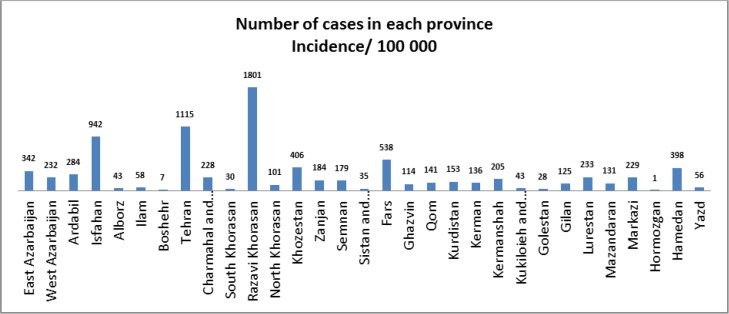
Incidence rate of human CE in different provinces of Iran during 1995–2014

Among 31 provinces of Iran, Razavi Khorasan Province from northeast was the more infected province with the 1801 CE cases and the lowest rate of CE was reported from Hormozgan Province in the south part of the country with the only one case in 2009 (Fig. 2).

Table 1 shows the distribution of human CE in various geographical zones of Iran reported during 1995 – 2014.

**Table 1:** Distribution of human CE in different areas (North, East, West, South and Center) of Iran during 1995–2014

***Geographical locations***																				
	***1995***	***1996***	***1997***	***1998***	***1999***	***2000***	***2001***	***2002***	***2003***	***2004***	***2005***	***2006***	***2007***	***2008***	***2009***	***2010***	***2011***	***2012***	***2013***	***2014***
**North**	13	18	18	17	23	13	25	20	8	6	16	24	16	19	17	27	25	23	32	38
**East**	46	49	84	62	10	12	13	75	10	17	12	10	68	93	79	10	81	10	82	97
					8	7	8		5	5	6	1				9		8		
**West**	37	47	87	63	67	69	56	39	48	56	39	56	55	33	36	79	115	117	98	107
**South**	61	60	63	83	28	29	20	10	12	20	16	9	9	5	9	9	10	8	4	10
**Center**	44	41	33	27	21	25	23	29	24	15	17	16	99	11	10	28	40	35	28	30
	5	1	6	5	6	1	8	1	6	8	1	7		5	6	3	3	6	6	6
**Total**	**602**	**590**	**584**	**486**	**435**	**478**	**470**	**429**	**414**	**406**	**364**	**352**	**242**	**263**	**246**	**506**	**615**	**596**	**429**	**550**

### Epidemiological profile of CE in 2014

**Fig. 3:**
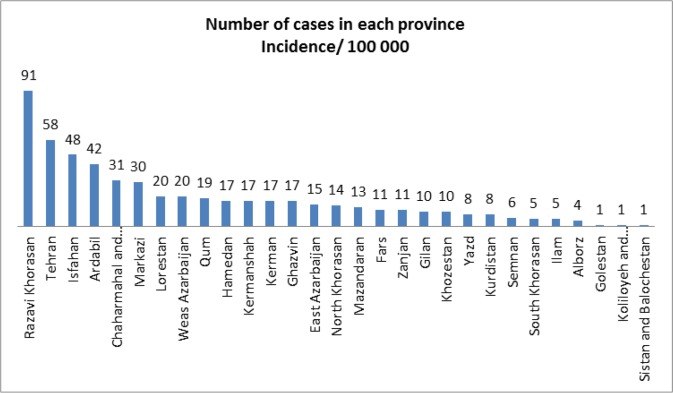
Incidence rate of human CE in different provinces of Iran in 2014

Fig. 3 illustrates the distribution of human CE in each province of the country in 2014. In that year, 550 cases were recorded. Razavi Khorasan, Tehran and Isfahan provinces had the highest incidence rates at 91, 58 and 48 cases, respectively.

**Fig. 4:**
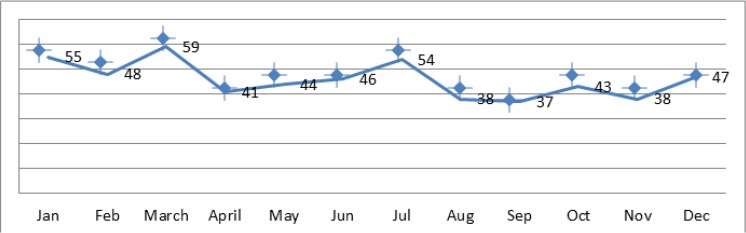
Number of cases of Hydatidosis in different months in 2014, Iran

**Fig. 5:**
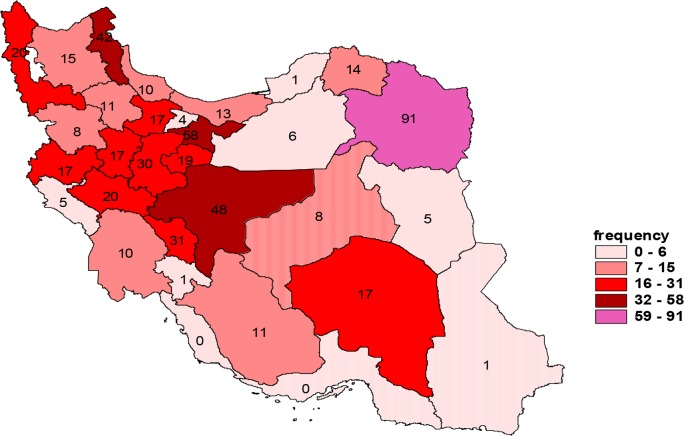
Distribution of human CE in different provinces of Iran, 2014

**Fig. 6:**
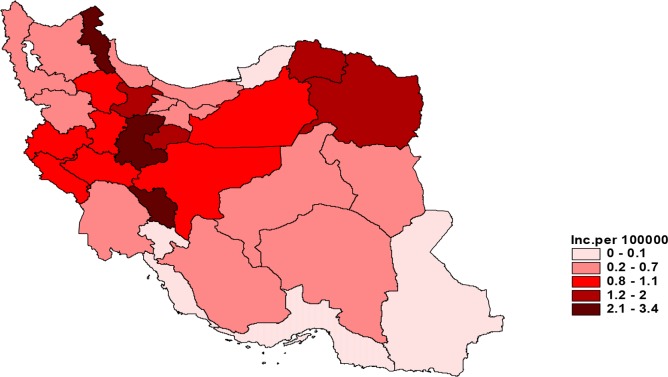
Distribution of Incidence of human CE in different provinces of Iran per 100000 inhabitants

According to, the highest annual incidence of new human CE in 2014 occurred in Mar, with 59 cases (10.72%), while the lowest rate was reported in Sep, with 37 cases (6.72%) (Fig. 4). Figures 5 and 6 show the distribution of human CE total cases and new cases of human CE in various parts of Iran.

**Fig. 7:**
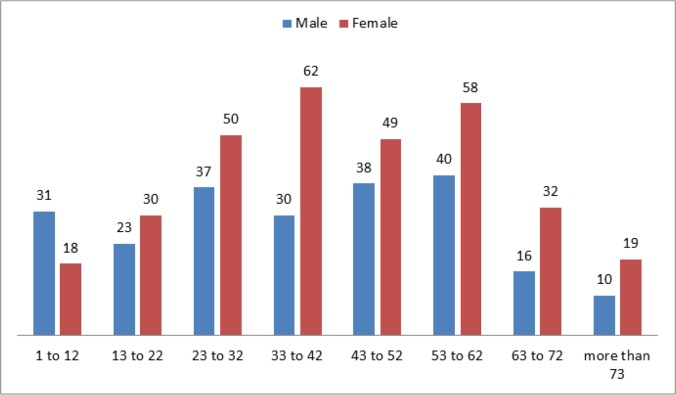
Distribution of human CE among different ages in Iran, 2014

Overall, 57%, 42% and 1% of patients were reported from Rural, urban and nomads’ areas, respectively. In current work, 60% of the vegetable were washed only by water. Detergents were used in 28% and disinfectants were used in 5% and 7% of vegetables were washed by all of them. Fig. 7 shows the distribution human CE among various age groups in all parts of Iran.

**Fig. 8:**
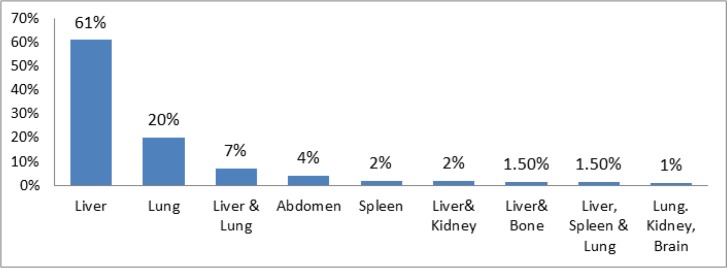
Distribution of human CE in different organs of patients in Iran, 2014

Liver, and lungs with the infection rate of 61% and 20%, respectively were the most involved organs (Fig. 8). Fifty-three percent of our cases had one cyst in their body. Two cysts and 3 cysts forming 18% and 7% of cases, respectively. In 8% of cases, number of cysts was more than 3 cysts and in 14% the number of cysts was unknown.

Three treatment options were used in our cases: surgery (in 41% of cases), drug treatment or chemotherapy (in 11% of cases) and mixed method (in 48% of cases). In this study, 52% of patients had no contact with dog and 48% have contact; of course, this difference was not statically significant. From all patients, 21 and 1 cases were from Afghanistan and Iraq, respectively.

## Discussion

WHO enumerates hydatidosis as one of the most serious global concern and has encouraged to focus on its prevention and control (13–15). However, due to the complex epidemiological aspects with various hosts and asymptomatic diseases, investigation, and control of the disease are very difficult. Human CE continues to be a significant public health problem in numerous countries. From several regions, there are alarming indications of increasing human health risks caused by echinococcosis(16).

*E. granulosus* has a global distribution and happens in all circumpolar, temperate, subtropical and tropical zones (17, 18). The prevalence of the parasite varies from sporadic to high and parts of Europe, Asia, Africa, Australia and South America have highest prevalence of *E. granulosus* (Fig. 4).

Current work indicated the trend in hydatidosis incidence in Iran from 1995 to 2014 and focused on the epidemiological position in 2014. We recorded an annual average of 8518 cases of hydatidosis over the two decades of the study and an annual average incidence of 18.96 per 100000 inhabitants. In Europe, northern and central areas have very low prevalence rates, regions of southern, south-eastern and eastern regions have medium or high prevalence rates and Iceland and Greenland are free of the parasite (18, 19). The annual incidence rates of human cases of cystic echinococcosis (CE) in some European countries varied between <1.0 and >8.0 per 100000 population. In Republic of China, the average annual incidence was 8.7 per 100000 in 1990(20).

The Ministry of Health of Turkey during 1987 and 1994 has recorded 21303 human cases of CE, with an annual incidence rate is 4.4 per 100000 inhabitants (21). In Our Northern neighbor, in Russia, during 15 yr (1983–1997) a total of 2863 cases of human Cystic echinococcosis was officially recorded (average of 191 cases per year) (22). 2.87% of Yemen human population was infected by Cystic echinococcosis(23). In 2014, 550 cases were recorded and Razavi Khorasan Province had the highest incidence rates at 91cases(24). Razavi Khorasan Province is neighbor with Pakistan and infected animal and foods may transfer the infection. In a study in Hyderabad, Pakistan recorded that 44 cases out of 43656 registered patients were polluted with cystic echinococcosis (25).

The highest morbidity rate of human CE was reported among 6 −20 years ages (3, 26). (3, 26). The most commonly affected age group in our survey was 33– 42 yr, followed by 53–62 yr. In New Zealand, patients with 25–44 yr of old were more infected with human cystic echinococcosis (22). Similarly, in Tasmania, 45–54 yr patients were more polluted (15, 27). Unlike to our study, the age group of less than 20 yr old higher rates of infection (5.0%); and the age group of 41–60 yr old have lower rate (1.33%) in Yemen (23). In another study in Uruguay, the age groups between 70 to 79 yr were most polluted than other age groups (28).

According to our results, human CE also affected slightly more females than males in most of age groups; but the difference was not statically significant. In a study in Yazd Province, 50% of the infected individuals were female and 50% were male (29). Similar to current work, in most series of individuals, their differences in the gender ratios of patients with CE were no significant (12, 26, 30). Of course, in a survey in Yemen all patients were female (23). The high infection rate in woman is probably due to more contact with dogs and because of the dogs being intense around the household where women spend most of their times.

According to geographical areas, because of the closer contact with dogs and low quality of education and health information, rural areas are usually more polluted than urban areas. CE happens most regularly in rural areas (31, 32). Over-all, 57% of patients were from rural areas, 1% was from nomads and others were from urban areas. The prevalence/incidence in urban areas is higher in compared to rural areas (33).

Liver and lung were the most ordinarily affected organs. Most of human cysts are clinically silent and are diagnosed accidentally or when complications befall (34). A study in Yazd Province showed that 75% of human hydatid cyst to be held in Liver (29). Reports indicate very rare incidence of the disease in other sites such as the heart, spleen, pancreas, and muscles (22). In our work, 4% and 2% of cysts formed in abdomen and spleen, respectively. The splenic contribution in hydatid disease is infrequent, routinely less than 2%–6% of all human Echinococcus cases. The infestation of the spleen commonly follows by two ways: 1: by arterial route through the hepatic and pulmonary filters, 2: rearward venous route (35, 36).

There are now three treatment choices for hydatid disease: surgery, which remains the most efficient treatment (37). Chemotherapy (secondary) is preferred where surgery is not accessible or when there are numerous cysts, and in unfeasible cases (38). In 3rd method, chemical drugs have also been used as an addition to surgery for prevention of spillage of cyst insides (39, 40). Because of the passive surveillance of data collection, some defects and limitations in data collection and report may happen.

## Conclusion

Human CE is still a major problem for health system of Iran and need to establish basic control programs. It is crucial to setting up standard diagnostic methods for early diagnosis, effective treatment, plan educational schedule for different social levels and control the diseases in different definitive and intermediate hosts.

## Ethical considerations

Ethical issues (Including plagiarism, informed consent, misconduct, data fabrication and/or falsification, double publication and/or submission, redundancy, etc.) have been completely observed by the authors.
